# Editorial: Classical and Novel Biomarkers for Cardiovascular Disease

**DOI:** 10.3389/fcvm.2022.943227

**Published:** 2022-06-15

**Authors:** Maria Perticone, A. Molfino, R. Maio

**Affiliations:** ^1^Department of Medical and Surgical Sciences, Magna Graecia University, Catanzaro, Italy; ^2^Department of Translational and Precision Medicine, Sapienza University, Rome, Italy; ^3^Azienda Ospedaliero-Universitaria Mater Domini, Catanzaro, Italy

**Keywords:** cardiovascular disease, biomarkers, cardiovascular event, screening, follow-up

Despite several efforts to prevent and treat atherosclerosis, cardiovascular disease (CVD) still remains a major cause of morbidity and mortality worldwide, and its prevalence is supposed to increase in the next years (1). In the last decades we observed a marked reduction in mortality rates for coronary heart disease (CHD) in Europe and United States of America, due to the improvements in acute care and prevention strategies; on the contrary, in the same period, both prevalence and mortality for CVD exponentially increased in low- and middle-income Countries, likely due to globalization (2).

In addition, CVD represents one of the most common comorbidities for several chronic diseases, including type 2 diabetes mellitus (T2DM), chronic kidney disease (CKD), and chronic obstructive pulmonary disease (COPD).

For these reasons, the use of biomarkers appears the most convenience option to screen and follow-up patients with CVD. The ideal biomarker should be widely available, low-cost and reliable. In the past decades several biomarkers have been proposed and used with this purpose (i.e., C-reactive protein, uric acid, troponin, natriuretic peptides, etc.) and several others are emerging (gamma-glutamyltransferase, miRNA, etc). The majority of these biomarkers are used to predict cardiovascular risk in specific settings of population.

To our knowledge, none of the existing biomarkers for cardiovascular disease are routinely used and scientifically validated among the general population and do not appear in cardiovascular risk scores. Thus, we proposed a Research Topic aimed to spread knowledge about useful novel biomarkers to predict and/or to manage cardiovascular risk, as well as to systematically analyze previously published data in order to draw robust conclusions on the use of specific biomarkers in the field of cardiovascular disease.

The Research Topic obtained a great success, since we received 28 submissions of which 15 have been published. Among these, we received 13 original articles and two reviews. The Research Topics of the published articles covered many of the most important and prevalent CVD, from heart failure, to light chain cardiac amyloidosis, pulmonary hypertension, acute myocardial infarction, etc.

The Research Topic collected both animal and human studies. In particular, Zaky et al. investigated the association of different echocardiographic, biochemical, and electrocardiographic correlates with progressive pulmonary arterial hypertension in rats. They demonstrated that, beyond classical invasive procedures used to assess myocardial and pulmonary vascular structure and function, also non-invasive methods (i.e., electrocardiogram and echocardiogram) can be used. Among biochemical biomarkers, authors identified plasma myosin light chain, cardiac troponin T, and fatty acid-binding protein-3 as useful tools for monitoring pulmonary hypertension progression. On the contrary, Zhao et al. demonstrated, in a population of 232 patients affected by idiopathic pulmonary arterial hypertension, that echocardiographic attenuated right heart remodeling is an independent non-invasive determinant of prognosis in this setting.

Remaining in the field of pulmonary hypertension, Zhang et al. demonstrated the role of CA125 as a new predictor of clinical impairment in patients with pulmonary hypertension and their results allowed to establish a cut-off value of CA 125 > 35 U/ml that is associated with 2-folds increased risk of 1-year clinical worsening. CA 125 has also been associated with poor prognosis in patients with light-chain cardiac amyloidosis, as demonstrated in the study by Li et al. In particular, the authors found that patients with high levels of CA 125 had a median overall survival time of 5 months, compared with the 25 months of patients with normal values of CA 125. Of note, the ROC curve showed that the prediction accuracy of CA 125 was not inferior to that of cardiac troponin T, N-terminal pro-B-type natriuretic peptide and LDH in patients with light-chain cardiac amyloidosis.

Another rare disease, such as dilated cardiomyopathy, has been studied by Ma et al.; in this study the authors utilized the Robust Rank Aggregation method to identify expressed genes responsible of dilated cardiomyopathy, allowing to identify a 7-gene signature predictive of dilated cardiomyopathy.

Another important Research Topic addressed in this collection is the cardiovascular risk assessment of hemodialysis patients. Hwang et al. investigated the role of circulating neprilysin as a biomarker of cardiovascular events in a wide population of hemodialysis patients. The authors demonstrated that the cumulative event rate of cardiac events was higher in patients in the 3rd tertile of neprilysin values; in addition, circulating neprilysin resulted as an independent predictor of cardiovascular events in this setting of patients, opening new scenarios on the use of this molecule as a biomarker of cardiovascular events in hemodialysis patients. A study conducted by Kim et al. in the same population demonstrated the role of another molecule, vascular adhesion protein-1 (VAP-1), as a predictor of cardiovascular events in this setting of patients. Taken together, these studies represent an important step in the cardiovascular risk stratification of hemodialysis patients.

When considering coronary artery disease, two original research articles have been published in this Research Topic; the paper by Le et al. focused on the role of conventional indicators of lipid oxidation, in particular of oxylipins, in discriminating among the number of diseased coronary arteries and in predicting 5-year outcomes in symptomatic patients. Interestingly, the authors found that a panel of five oxylipins allows to diagnose three diseased arteries with 100% sensitivity and 70% specificity, thus helping clinicians in the diagnostic and therapeutic process of coronary artery disease. Similarly, the study by Yifan et al. investigated the role of ferroptosis (a novel type of programmed cell death and marker of cell injury usually studied in cancer development) as a biomarker of myocardial cell injury in circulating endothelial cells; they concluded that ferroptosis-related genes might be used as potential specific diagnostic markers for myocardial infarction. Remaining in the setting of acute cardiovascular disease, Yang S. et al. demonstrated that Neutrophil Extracellular Trap Levels show a significant diagnostic and predictive value of disease severity in patients with acute aortic dissection, while Zhai et al. used plasma osmolarity to predict in-hospital cardiac mortality in cardiac intensive care unit patients; interestingly, they observed that both hyposmolarity and hyperosmolarity were independently associated with an increased in-hospital cardiac mortality (“*U*”-shaped relationship).

Of note, this Research Topics also included the results of a multicenter prospective study conducted by Mise et al., that demonstrated that the urinary excretion of high-mannose glycan may be a valuable marker for predicting cardiovascular events in type 2 diabetes mellitus patients.

The two reviews of the Research Topic were both focused on heart failure. In particular, the meta-analysis by Yang C. et al. evaluated the diagnostic value of soluble suppression of tumorigenicity (sST2) in heart failure, with non-conclusive results. The review by Janssen et al. investigated another potential application of natriuretic peptides in heart failure; in particular, the authors analyzed the prognostic value of circulating natriuretic peptides before left ventricular assist device implantation for all-cause mortality and major adverse events, concluding that natriuretic peptides levels are of limited value in patients selection for left ventricular assist device therapy.

Taken together, all these papers underline the need to have simple, cost-effective and easily available biomarkers for the diagnosis and follow-up of CVD. The wide number of biomarkers investigated in this Research Topic can help physicians now and in the next future to better manage CVD, highly impacting on patient's prognosis and quality of life.

## Author Contributions

MP, AM, and RM wrote the Editorial and approved the last version. All authors contributed to the article and approved the submitted version.

## Conflict of Interest

The authors declare that the research was conducted in the absence of any commercial or financial relationships that could be construed as a potential conflict of interest.

## Publisher's Note

All claims expressed in this article are solely those of the authors and do not necessarily represent those of their affiliated organizations, or those of the publisher, the editors and the reviewers. Any product that may be evaluated in this article, or claim that may be made by its manufacturer, is not guaranteed or endorsed by the publisher.
